# Assessing Cardiac Flow Measurements Using a Noninvasive Photoplethysmography-Based Device Compared to Invasive Pulmonary Artery Catheter

**DOI:** 10.1016/j.jacadv.2025.102093

**Published:** 2025-08-22

**Authors:** Dean Nachman, Arik Eisenkraft, Eldad Rahamim, Mahsati Ibrahimli, Asen Asenov, Nir Goldstein, Yotam Kolben, Segev Huly, Arik Ben Ishay, Meir Fons, Michael Tabi, Roei Merin, Offer Amir, Rabea Asleh

**Affiliations:** a Heart Institute, Hadassah Medical Center and Faculty of Medicine, Hebrew University of Jerusalem, Jerusalem, Israel; b Institute for Research in Military Medicine, Faculty of Medicine, The Hebrew University of Jerusalem and the Israel Defense Force Medical Corps, Petah Tikva, Israel; c Biobeat Technologies Ltd, Petah Tikva, Israel

**Keywords:** cardiac flow measurements, congestive heart failure, noninvasive monitoring, photoplethysmogram, remote patient monitoring, Swan-Ganz catheter

## Abstract

**Background:**

Invasive monitoring is considered the gold standard for hemodynamic monitoring, yet it poses inherent risks.

**Objectives:**

The aim of the study was to compare invasive hemodynamic measurements using a noninvasive wearable photoplethysmography-based (PPG) monitor and an invasive pulmonary artery catheter.

**Methods:**

Heart failure patients undergoing right heart catheterization were recruited. The PPG-based monitor was applied, and readings of cardiac output (CO) and systemic vascular resistance (SVR) commenced in parallel to invasive hemodynamic measurements and CO calculations using indirect Fick’s (IF) and thermodilution (TD) methods. Bland-Altman plots and Pearson correlations were used to assess the accuracy and agreement between the techniques. Limits of agreement below ±30% compared to TD were regarded as highly concordant.

**Results:**

A total of 90 patients (56.6% [51/90] men, mean age 62 ± 16 years) were included in the final analysis. The limits of agreement and bias (lower/upper limit of 95% CI) for CO and SVR were −19.1/14.5%, −2.3% (−4.2/−0.4%) and −27.4/12.3%, −7.5% (−9.7/−5.2%) for the PPG compared to the TD; −28.6/29.8%, 0.5% (−2.7/3.8%) and −42.3/20.5%, −10.9% (−14.4/−7.3%) for the PPG compared to IF method; and −46.1/34.1%, −6.0% (−10.6/−1.3%) and −26.8%/58.3%, 15.7% (10.7/20.7%) for the TD compared to the IF method. Pearson coefficients (lower/upper limit of 95% CI) between the PPG method and the TD, the PPG method and IF, and the TD and IF were 0.959 (0.938/0.974), 0.844 (0.767/0.897), and 0.775 (0.668/0.851) for CO, and 0.936 (0.902/0.959), 0.865 (0.796/0.911) and 0.687 (0.546/0.79) for SVR, respectively. Similar correlations and biases were found with different BMIs and skin color tones.

**Conclusions:**

The PPG-based device correlates well with invasive methods. (Comparing Cardiac Output Measurements Using a Wearable, Wireless, Non-invasive PPG-Based Device to a Swan Ganz Catheter; NCT04955184)

Invasive monitoring by pulmonary artery catheter (PAC) is considered the gold standard of hemodynamic monitoring and may be required in seriously ill intensive care unit patients, providing important indications of physiological changes and early recognition of patient deterioration.^1^^,^^2^ Nonetheless, the invasive nature of these procedures carries inherent risks.^2^, ^3^, ^4^, ^5^ Adoption of less invasive technologies could diminish the frequency of complications and potentially broaden the scope of advanced hemodynamic surveillance to include patients in general wards and even on an outpatient basis.

Emerging technologies such as volume-clamp or bioimpedance with pulse-contour analysis have introduced devices that allow for minimally or noninvasive hemodynamic monitoring, presenting potential substitutes to traditional methods.^6^^,^^7^ However, these innovative devices demonstrate a significant variance in accuracy compared to invasive techniques, and their precise application in clinical practice remains to be conclusively determined.^8^, ^9^, ^10^, ^11^, ^12^

The critical role of invasive hemodynamic monitoring in the management of cardiac patients, across both emergency and routine care contexts, cannot be overstated. The clinical imperative lies in identifying noninvasive, user-friendly modalities for advanced monitoring that can be adopted broadly. This study aims to evaluate a photoplethysmography (PPG)-based wearable monitoring device, which has shown promise in preliminary validations for tracking advanced hemodynamic parameters.^13^^,^^14^ In this study, we have evaluated the measurements of the PPG-based wearable advanced hemodynamic monitor against the gold standard PAC measurements in patients undergoing evaluation in a heart failure unit, focusing on measurements of cardiac output (CO) and systemic vascular resistance (SVR).

## Methods

### General and ethical considerations

This prospective, comparative clinical trial has received the requisite ethical clearance from the Institutional Review Board at Hadassah Medical Center in Jerusalem, Israel (0219-21-HMO), and was registered in the clinical trial registry (NCT04955184). Prior to their inclusion in the study, all participants were required to provide written informed consent, confirming their voluntary participation and their understanding of the study's procedures and objectives.

The PPG-based monitoring devices utilized in the study were supplied by the manufacturer at no cost, alongside technical support that was furnished by the company's representatives. The company's personnel did not exert any influence over the experimental design or the interpretation of the data.

### Study population

Ambulatory patients aged 18 years and above, who were scheduled to undergo right heart catheterization for clinical evaluation, were included as participants. The exclusion criteria for the study included pregnant women, individuals diagnosed with cognitive impairment or mental disorders, and employees of the institution in which recruitment was conducted.

### The PPG-based wearable device

The PPG-based medical-grade patch-monitor device (BB-613WP, Biobeat Technologies Ltd) was previously described.^14^^,^^15^ Shortly, it includes a reflective PPG sensor providing multiple vital signs using the pulse wave transit time approach (Central Illustration). Monitoring started following a baseline calibration process, in which the average of three blood pressure measurements taken with an Food and Drug Administration-cleared cuff-based blood pressure device is obtained. When in routine clinical use, the calibration of the PPG-based device is required once every 3 months, or when a significant change in the hemodynamic status has occurred, for example, following an intervention. In this study, calibration was performed using the Mennen Medical Horizon XVu Hemodynamic NIBP Monitoring device (Mennen Medical Ltd.). Following the calibration phase, the study continued with parallel measurements. At the time of the study, the device was FDA-cleared for measurements of cuffless noninvasive blood pressure, SpO_2_, pulse rate, respiratory rate, and temperature. There was no need for further calibration of the PPG-based devices following the initial baseline measurements, as calibration should be performed once every 3 months. Designated gateways were deployed and installed in the catheterization lab to ensure continuous monitoring, data transmission, and automatic data collection of all measurements. In this study CO and SVR were repeatedly measured using the PPG-based device in parallel to the invasive measurements using time marks in the data log.

### Study protocol

Upon securing informed consent, participants were requested to provide demographic data and a comprehensive medical history via a structured questionnaire. Anthropomorphic data, including weight (in kilograms), height (in meters), and skin tone (Fitzpatrick scale), were collected. The next step involved the application of the PPG-based monitoring device, which was affixed to the skin at the upper left sternal margin of the chest. Prior to initiation of the monitoring process, the device was carefully calibrated against a standard cuff-based blood pressure apparatus to ensure accuracy. The PPG-based device commenced intermittent monitoring at five-second intervals as the subjects were admitted into the catheterization laboratory.

Subsequently, a Swan-Ganz PAC (Edwards LifeSciences; with Cordis 7F introducer) was inserted into the pulmonary artery using fluoroscopic guidance. The PAC permitted on-the-spot hemodynamic assessments, employing both the indirect Fick’s (IF) principle and thermodilution (TD) techniques. Throughout the procedure, continuous parallel monitoring was performed, juxtaposing the data from the invasive PAC with the noninvasive PPG-based device.

The attending medical personnel remained blinded to the readings acquired via the noninvasive monitoring device during the procedure to prevent any observer bias and were not involved in the data analysis. A comprehensive comparison and analysis of the gathered data from both the invasive and noninvasive monitoring methods was conducted retrospectively, subsequent to the completion of the data collection phase. This approach ensured objective evaluation of the PPG-based device’s performance in a clinical setting.

### Cardiac output measurements using indirect Fick

Arterial and mixed venous blood samples were simultaneously obtained via the arterial line and the distal (pulmonary arterial) opening of the PAC, respectively, for the determination of PO_2_, PCO_2_, pH, base excess, and (calculated) SaO_2_ (ABL 520; Radiometer). If an arterial line was not clinically indicated, pulse oximetry readings were used. The arterial and mixed venous oxygen contents (CaO_2_ and CvO_2_) were calculated as the product of hemoglobin (g/L), the hemoglobin-binding constant for oxygen (1.34 g/L), and oxygen saturation. VO_2_ was estimated as 125 mL/min/m^2^. Stroke volume (SV) and SVR were then calculated accordingly.

### Cardiac output measurements using thermodilution

Ten milliliters of 0 to 4 °C saline solution was manually injected into the right atrium through the PAC at the end of expiration. Blood temperature was measured by a thermistor at the tip of the PAC within the pulmonary artery, providing the TD profile, and the process was repeated between 3 and 5 times for each subject; tracings differing by >10% were discarded.^16^

### Systemic vascular resistance calculation

Since in most patients, there was no indication for an arterial line, mean arterial pressure from cuff-based measurement was utilized with a corresponding right atrial pressure measured invasively to calculate SVR with the following equation: SVR = (mean arterial pressure - right atrial pressure)/CO.

### Statistical analysis

The final analysis includes comparisons that were performed between the IF measurements and the measurements of the TD measurements, taken up to 10 minutes after the IF measurements, and between the IF measurements and the PPG-based measurements taken up to 3 minutes after the IF measurements as long as the clinical parameters remained stable.

In the statistical analysis of this study, the Bland-Altman method was used to evaluate the level of agreement between different measurement methodologies for CO, SV, and SVR. For each comparative analysis, we calculated the mean difference (bias) and the 95% limits of agreement (LOA), in addition to the percentage difference, to quantitatively assess the concordance between methods. The accepted cutoff of LOA below ±30% compared to TD was regarded as highly concordant.^17^ Pearson correlation coefficient was employed to ascertain the degree of linear relationship between TD or IF method and PPG measurements for CO and SVR, as well as between TD and the IF method. Further, the analysis was refined by stratifying the data according to body mass index and skin color tone following the Fitzpatrick color scale to explore any potential variations in measurement accuracy related to these specific demographic and physiological factors.^18^ In short, the Fitzpatrick color scale is an accepted numerical classification for human skin color as a way to estimate the response of different types of skin to ultraviolet light, ranging from 1-very light or white, always burns and never tans, up to 6-very dark, deeply pigmented, and never burns.

## Results

### Patient characteristics

The demographic and medical background (Table 1) of the 90 participants enrolled in this study presents a cohort with a mean age of 62 ± 16 years, predominantly male (56.6%, 51/90). The average body mass index was observed to be 30 ± 7 kg/m^2^, indicating a patient population with a tendency toward overweight or obesity. Cardiovascular parameters suggest a varied range of cardiac function among participants, with an average ejection fraction of 45% ± 14%, heart rate of 81 ± 16 beats/min, and CO of 5 ± 1 L/min. Nearly one-third (29/90) of the participants exhibited a reduced ejection fraction of 40% or below, while the majority (53.3%, 48/90) had an ejection fraction above 50%. The medical history of the cohort revealed a high prevalence of hypertension and type 2 diabetes mellitus, each affecting 54.4% (49/90) of the subjects.

**Table 1:** General Demographic, Cardiovascular, and Medical History Details of the Participants (N = 90)

Age (y, average ± SD)	62 ± 16
Males (%)	51 (56.6%)
BMI (kg/m^2^, average ± SD)	30 ± 7
Cardiovascular parameters, average ± SD	
Ejection fraction (%)	45 ± 14
Ejection fraction ≤40% (n = 29)	27 ± 8
Ejection fraction 41%-49% (n = 13)	44 ± 2
Ejection fraction ≥50% (n = 48)	56 ± 5
Heart rate (beats/min)	81 ± 16
Stroke volume (mL)	62 ± 17
Systolic blood pressure (mm Hg)	142 ± 31
Diastolic blood pressure (mm Hg)	80 ± 14
Hemodynamic parameters, median (25th/75th percentiles)	
Cardiac output (L/min)	5.1 (4.1/6.1)
Cardiac output <4 L/min (n = 21)	
Cardiac output 4-8 L/min (n = 64)	
Cardiac output >8 L/min (n = 3)	
CVP (mm Hg)	8 (4/13.3)
SVR (dynes/s/cm^5^)	1,589 (1,202.2/1712.5)
PVR (dynes/s/cm^5^)	168.9 (120.1/236.6)
LAP (PCWP) (mm Hg)	17.5 (10.7/24.2)
MPAP (mm Hg)	29 (20.7/37)
Medical history, n (%)	
Atrial fibrillation	15 (16.6%)
Connective tissue diseases (APLA, amyloidosis, and sarcoidosis)	9 (10%)
CRF (including ESKD, dialysis, and kidney transplant)	17 (18.8%)
HTN	49 (54.4%)
Hyperlipidemia (including dyslipidemia)	27 (30%)
IHD (including MI and PTCA)	18 (20%)
LVAD	2 (2.2%)
Moderate/severe valvular disease	6 (6.6%)
Myocarditis	5 (5.5%)
OSA	9 (10%)
Pulmonary HTN	11 (12.2%)
T2DM	49 (54.4%)
Medications, n (%)	
ACEi/ARB/ARNI	40 (44.4%)
Anticoagulation	6 (6.6%)
Antiplatelet	26 (28.8%)
Beta-blocker	31 (34.4%)
Biguanides	21 (23.3%)
Corticosteroids	12 (13.3%)
Digoxin	3 (3.3%)
Insulin	12 (13.3%)
Loop diuretic	48 (53.3%)
MRA	33 (36.6%)
Prostaglandin analogs	2 (2.2%)
Proton pump inhibitors	40 (44.4%)
SGLT2 inhibitor	17 (18.8%)
Statin	43 (47.7%)
Thiazide	3 (3.3%)
Thyroid hormone drugs	9 (10%)
Xanthine oxidase inhibitors	17 (18.8%)

ACEi = angiotensin-converting enzyme inhibitor; APLA = antiphospholipid antibody; ARB = angiotensin II receptor blocker; ARNI = angiotensin receptor-neprilysin inhibitor; BMI = body mass index; CRF = chronic renal failure; CVP = central venous pressure; ESKD = end-stage kidney disease; HTN = hypertension; IHD = ischemic heart disease; LAP = left atrial pressure; LVAD = left ventricular assist device; MI = myocardial infarction; MPAP = mean pulmonary artery pressure; MRA = mineralocorticoid receptor antagonist; OSA = obstructive sleep apnea; PCWP = pulmonary capillary wedge pressure; PTCA = percutaneous transluminal coronary angioplasty; PVR = pulmonary vascular resistance; SGLT2 = sodium-glucose cotransporter-2; SVR = systemic vascular resistance; T2DM = type 2 diabetes mellitus.

A single IF method measurement was taken for each patient. In 7 patients, IF measurements were not calculated due to technical problems with the blood gas machine. An outlier measurement was found in the TD measurements, and in 4 patients, the TD measurements were taken more than 10 minutes following the IF measurements. In two patients, the PPG-based patch was not properly placed. These measurements were excluded from the final analysis, resulting in 81 comparisons between IF and PPG, 78 comparisons between IF and TD, and 82 comparisons between PPG and TD.

When looking at the low and high limits of the PPG-based device measurements recorded in this study, we found SV ranged from 17.6 to 111.9 mL/beat, CO ranged from 1.5 to 13.2 L/min, cardiac index (defined as CO divided by the body surface area) ranged from 1.2 to 4.2 L/min/m^2^, and SVR ranged from 786 to 3,880 dyn/s/cm^5^.

### Cardiac output

**Figure 1:**
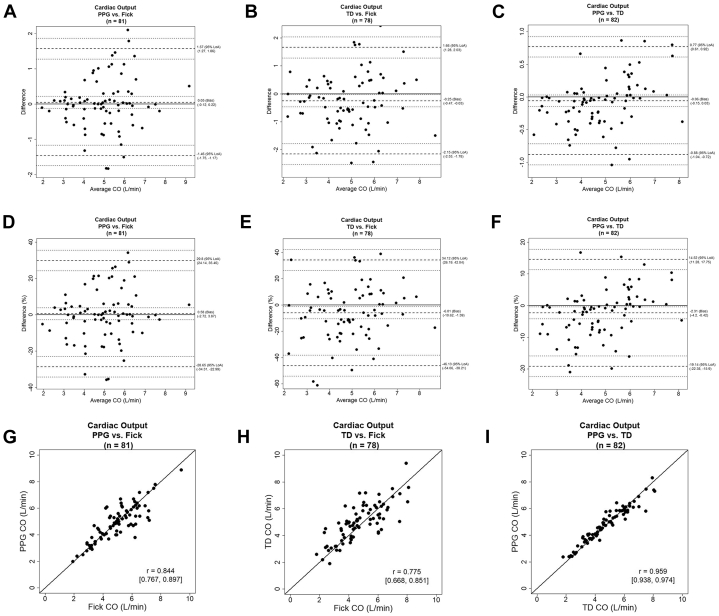
**Comparison of Cardiac Output Measurements** (A) Bland-Altman analysis between the PPG-based device and the indirect Fick method; (B) Bland-Altman analysis between the TD method and the indirect Fick method; (C) Bland-Altman analysis between the PPG-based device and the TD method; (D) percentage of difference of the PPG-based device and indirect Fick; (E) percentage of difference of the TD and indirect Fick; (F) percentage of difference of the PPG-based device and the TD method; (G) the correlation between the PPG-based device and the indirect Fick method; (H) the correlation between the TD method and the indirect Fick method; and (I) the correlation between the PPG-based device and the TD method. LoA = limit of agreement; PPG = photoplethysmography; TD = thermodilution.

The mean ± SD value of CO measured using the IF method was 5.0 ± 1.4 L/min, and the mean values with the PPG-based device and the TD method measured in parallel were 4.9 ± 1.3 L/min and 4.9 ± 1.6 L/min, respectively. Bland-Altman analysis of CO showed a lower/upper LOA and bias (lower/upper limit of 95% CI) of −1.4/1.5 L/min, −0.05 (−0.1/0.2) L/min for PPG vs IF, −2.1/1.6 L/min, −0.2 (−0.4/-0.0) L/min for TD vs IF, and −0.8/0.7 L/min, −0.06 (−0.15/0.0) L/min for the PPG vs TD (Figures 1A to 1C). When calculating percentage of difference, we found a lower/upper LOA and bias (lower/upper limit of 95% CI) of −28.6/29.8%, −0.5(-2.7/3.8)% for the PPG vs IF, −46.1/34.1%, −6.0 (-10.6/1.3)% for the TD vs IF, and −19.1/14.5%, −2.3 (-4.2/-0.4)% for the PPG vs TD, respectively (Figures 1D to 1F). Pearson coefficients (lower/upper limit of 95% CI) were 0.844 (0.767/0.897) for PPG vs IF, 0.775 (0.668/0.851) for TD vs IF, and 0.959 (0.938/0.974) for PPG vs TD (Figures 1G to 1I). The data are summarized in a table provided in the (Supplemental Table 1).

### Systemic vascular resistance

**Figure 2:**
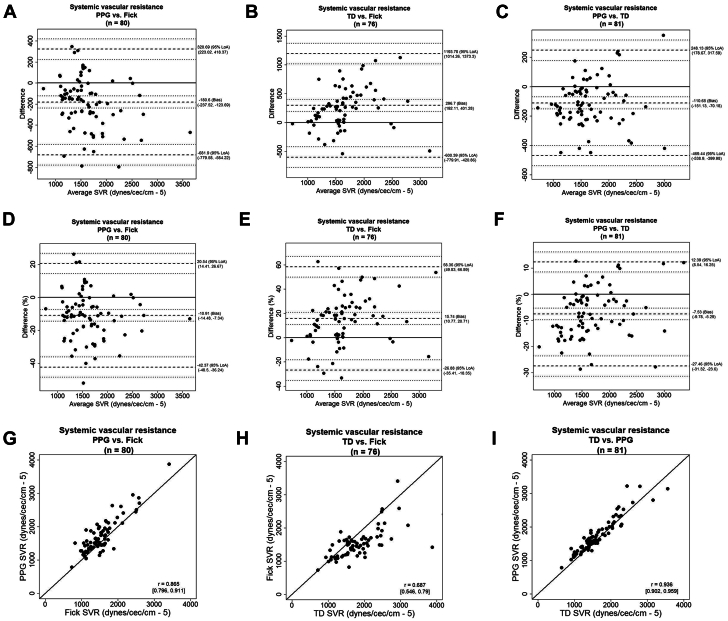
**Comparison of Systemic Vascular Resistance Measurements** (A) Bland-Altman analysis between the PPG-based device and the indirect Fick method; (B) Bland-Altman analysis between the TD method and the indirect Fick method; (C) Bland-Altman analysis between the PPG-based device and the TD method; (D) percentage of difference of the PPG-based device and indirect Fick; (E) percentage of difference of the TD and indirect Fick; (F) percentage of difference of the PPG-based device and the TD method; (G) the correlation between the PPG-based device and the indirect Fick method; (H) the correlation between the TD method and the indirect Fick method; and (I) the correlation between the PPG-based device and the TD method. SVR = systemic vascular resistance; other abbreviations as in Figure 1.

The mean values of the SVR measured using the IF method (for CO calculation), the PPG-based device, and the TD method were 1720 ± 513 dyn/s/cm^5^, 1737 ± 511 dyn/s/cm^5^, and 1793 ± 603 dyn/s/cm^5^, respectively. Bland-Altman analysis of SVR showed a lower/upper LOA and bias (lower/upper limit of 95% CI) of −681.9/320.6 dyn/sec/cm^5^, −180.6 (−237.5/−123.6) dynes/sec/cm^5^ for PPG vs IF, −600.3/1,193.7 dyn/sec/cm^5^, 296.7 (192.1/401.2) dynes/sec/cm^5^ for TD vs IF, and −469.4/248.1 dyn/sec/cm^5^, −110.6 (−151.1/−70.1) dynes/sec/cm^5^ for the PPG vs TD (Figures 2A to 2C). When calculating percentage of difference, we found a lower/upper LOA and bias (lower/upper limit of 95% CI) of −42.3/20.5%, −10.9 (−14.4/−7.3)% for the PPG vs IF, −26.8/58.3%, 15.7 (10.7/20.7)% for the TD vs IF, and −27.4/12.3%, −7.5(−9.7/−5.2)% for the PPG vs TD, respectively (Figures 2D to 2F). Pearson coefficients (lower/upper limit of 95% CI) were 0.865 (0.796/0.911) for PPG vs IF, 0.687 (0.546/0.79) for TD vs IF, and 0.936 (0.902/0.959) for PPG vs TD (Figures 2G to 2I). The data are summarized in a table provided in the (Supplemental Table 2).

**Central Illustration:**
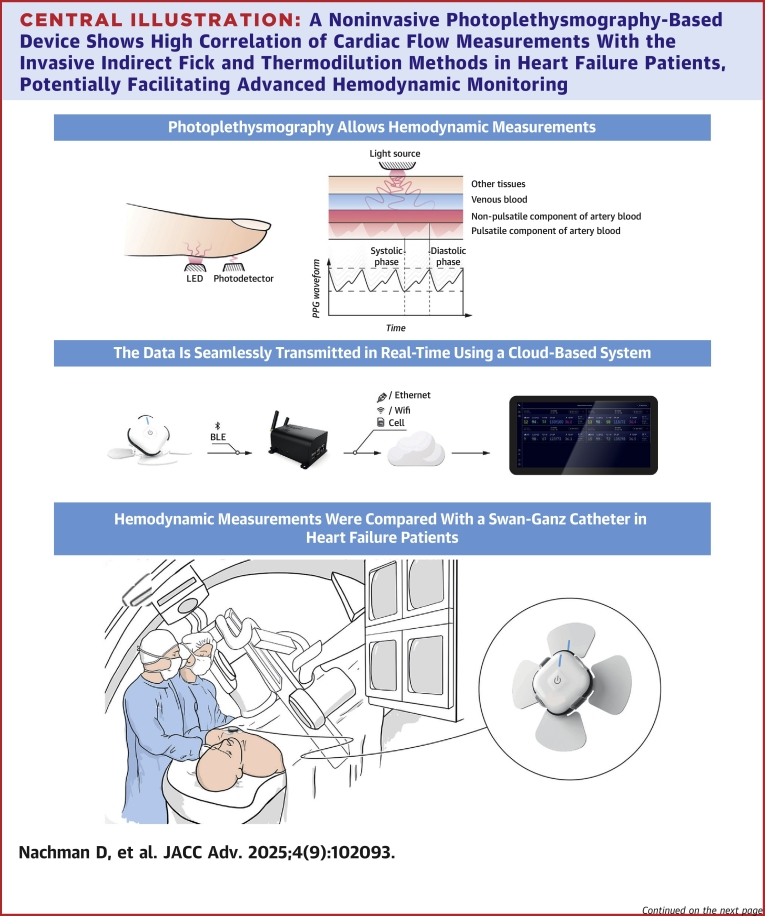

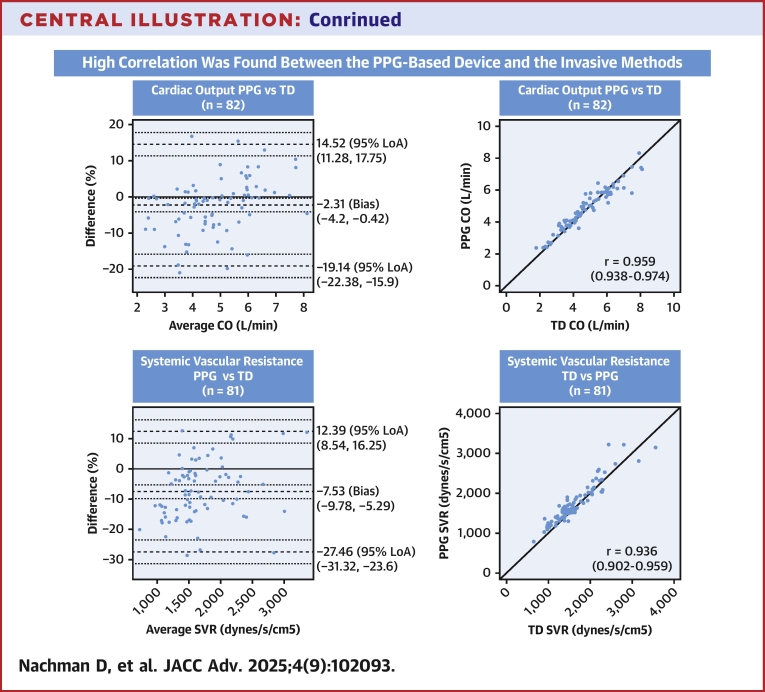
**A Noninvasive Photoplethysmography-Based Device Shows High Correlation of Cardiac Flow Measurements With the Invasive Indirect Fick and Thermodilution Methods in Heart Failure Patients, Potentially Facilitating Advanced Hemodynamic Monitoring** CO = cardiac output; other abbreviations as in Figures 1 and 2.

### Stratification based on skin color and body mass index

Subgroup analysis, delineating results by body mass index and skin tone, exhibited congruent outcomes to all participants’ analysis when evaluated through Bland-Altman plots and Pearson correlation. These findings substantiate the consistency of the device's performance across diverse physiological profiles. Visual representations of these results are appended (Supplemental Figures 1 to 10), where specific data points and statistical measures are annotated within each figure for clarity.

## Discussion

Invasive monitoring methods provide continuous measurement of numerous cardiopulmonary parameters, yet they are time-consuming and prone to complications.^2^^,^^3^^,^^19^ Moreover, invasive methods are technically demanding and usually necessitate an intensive care unit stay, limiting their use to specific high-risk patients.^20^ Thus, advanced hemodynamic monitoring by noninvasive means might enable thoughtful diagnosis and treatment of unstable patients and expand the utilization of such monitoring to stable and even ambulatory patients while minimizing morbidity and mortality and maximizing clinical outcome. Moreover, by eliminating the need for disposables, catheterization labs, intensive care monitoring specialists, and associated complications, PPG-based monitoring has the potential to substantially reduce per-patient costs compared with invasive PAC techniques. The simple, wearable form factor and minimal training requirements of PPG-based monitoring also facilitate seamless integration into both inpatient and ambulatory workflows, underscoring its real-world feasibility for broad clinical adoption.

When it comes to examining the reliability of a device for measuring advanced hemodynamic parameters, the value of LOA of ±30% for measuring CO compared to an invasive method, usually TD, published in 1999 by Critchley and Critchley, is still accepted in the literature and is widely used in many comparisons.^4^^,^^17^^,^^21^, ^22^, ^23^, ^24^, ^25^, ^26^ Though relatively accepted, Peyton and Chong provided a meta-analysis of papers comparing minimally invasive CO monitors to TD, with several important statements regarding the Critchley and Critchley manuscript.^8^^,^^17^ Based on their updated analysis and assessment, they have stated that TD has no better precision than the other methods surveyed during conditions of unstable hemodynamics and that the arbitrary ±30% criterion is questionable. In their meta-analysis, the methods they have looked at achieved LOA that were very similar. As the various methods are based on quite different physical and physiological principles, this suggests that a fundamental limitation exists to the precision of agreement with a given reference standard like TD that can be achieved in clinical practice. Moreover, it is independent of the particular method being tested. They also state that this level of precision of agreement remains well outside the ±30% limits across a range of patient groups and clinical situations. Based on their empirical findings, LOA with TD of 45% represents a more realistic expectation of achievable precision in clinical practice.

The prevailing discrepancies in the literature regarding validation standards for noninvasive hemodynamic monitoring necessitated a different methodical approach in our evaluation and validation of the PPG-based device. Initially, we adopted the widely accepted agreement threshold of ±30% against TD as our benchmark for assessing the PPG-based device's performance. Moreover, in the absence of official validation criteria for noninvasive advanced hemodynamic monitors, we compared the PPG-based device against two established invasive techniques: TD and the IF method. This comparison was further extended to an intermethod analysis between TD and IF to refine our understanding of the realistic limits of agreement. Lastly, due to the lack of established validation thresholds for other advanced hemodynamic indices, our study utilized analogous criteria for SVR as those applied for CO.

In evaluating the study's outcomes against the aforementioned criteria, the analysis revealed that the LOA for CO measurement between the PPG-based device and TD were consistently below the 30% threshold, with values between −19.1% and −14.5% (Figure 1). Similarly, when compared with the IF method, the LOA remained under 30%, indicating a substantial correlation with the invasive measurements. Notably, the agreement limits between the two invasive techniques themselves surpassed the 30% mark (−46.1%, 34.1%). These findings are in concordance with previous comparative studies of these methods that found estimated error as high as 40 to 65%, setting the clinically acceptable LOA.^25^^,^^27^, ^28^, ^29^ The results suggest that the noninvasive PPG-based device is comparably effective to the established invasive methods currently in use for both research and clinical applications.

Further analysis of the SVR measurements yielded a similar coherent pattern (Figure 2). The LOA for comparisons between the PPG-based device and TD remained beneath the ±30% threshold. In contrast, all comparisons between the IF method and TD exceeded the ±30% threshold and were notably higher than those observed when comparing the PPG-based device with each of the invasive methods. These findings suggest the potential for a broader applicability of the PPG-based measurements in hemodynamic monitoring.

As extensively elaborated in the introduction, despite numerous commercial and academic endeavors to devise noninvasive techniques for CO measurement, the accuracy and reliability of these methods have been inconsistent and did not conform with the ±30% LOA criterion, rendering them currently unsatisfactory for clinical application. Studies comparing noninvasive CO monitoring technologies, including endotracheal bioimpedance, finger-cuff pulse-contour, applanation tonometry, thoracic bioimpedance, pulse-transit time analysis, and CO_2_-rebreathing against pulmonary artery TD in elective cardiac surgery patients, all report percent errors of 35 to 58% and poor trending, well above the 30% threshold for clinical reliability.^7^^,^^12^ Furthermore, the practical deployment of such instrumentation has proven to be operationally challenging.^7^^,^^8^^,^^25^^,^^30^, ^31^, ^32^ In the current experiment, the PPG-based device exhibited superior validity and accuracy in measuring CO compared to previously assessed devices, and this was also reflected across a range of other hemodynamic parameters.

As detailed in the introduction section, this technology has been evaluated in several studies in the past, showing similar results to what was found in the current evaluation, with LOA as low as 25% and a small bias (0.42 L/min) compared to PAC in a swine model of controlled hemorrhagic shock.^14^ The PPG-based device also demonstrated high correlations of CO measurements compared with pulse contour CO in a small number of septic patients in the general intensive care unit.^13^ The results of the current study add more to the confidence in utilizing PPG-based devices for monitoring advanced hemodynamic parameters, including CO and SVR. These parameters are traditionally measured using invasive PAC, which is regarded as the gold standard for hemodynamic assessment. Therefore, the results indicate that PPG-based devices accurately measure these hemodynamic parameters, which are essential for patient monitoring and treatment.

In light of previous reports on the lower validity and accuracy of PPG-based devices in measuring arterial oxygen saturation in darker skin tones and inaccurate measurements in patients with overweight and obesity, a comparison of the results was made according to these subgroups. It was found that in all the subgroups stratified based on skin tone and body mass index, the results still showed high validity and accuracy that met the criteria set forth. These findings contribute to the generalizability of the research results to diverse population groups.

Lastly, the participants in this study had multiple cardiovascular conditions or conditions that are related to acute or chronic cardiovascular morbidity and represent the entire spectrum of ejection fractions and comorbidities as depicted in Table 1. Also, most ambulatory patients were chronically treated with various medications targeting their underlying heart failure, as well as other medications directed at other chronic medical conditions. This study population is similar to the prevalent cardiovascular diseases among heart failure patients as they appear in the literature.^33^^,^^34^ Taken together, the patient population included in this study is representative of ambulatory and hospitalized heart failure patients, making the findings applicable to patients in such care settings.

### Study limitations

The principal limitation of this study lies in the episodic nature of the measurements; the longitudinal validity of serial assessments over extended durations or through significant hemodynamic shifts remains unexplored. Moreover, despite the inclusion of participants exhibiting a spectrum of CO, including a small number of patients with notably low and high values, most subjects maintained hemodynamic stability throughout the evaluation period. Additionally, the study did not include comparisons across varied hemodynamic states, as seen in patients with veno-arterial fistulas or with septic shock. Measurements were also exclusively conducted on supine, stationary patients. Given the anticipated use of this monitoring technology in ambulatory settings, further studies are warranted to assess its reliability in mobile patients and under different clinical conditions. Including patients with varying hemodynamic states (eg, septic shock and severe hypotension) would provide a more comprehensive validation.

## Conclusions

The PPG-based monitoring device provides CO and SVR measurements that correlate well with the gold standard invasive methods. Subjected to further studies, this technology holds the potential for application in complex clinical scenarios where invasive monitoring is less desirable or available. It may further broaden the scope of advanced hemodynamic monitoring to include ambulatory patients, thereby enhancing the precision and personalization of care.

Perspectives **COMPETENCY IN PATIENT CARE:** Among heart failure patients undergoing right heart catheterization, a noninvasive PPG-based device provided cardiac flow measurements that correlated well with the invasive methods, with no adverse events related to its use.

**TRANSLATIONAL OUTLOOK:** Accurate hemodynamic monitoring has an important role in both prehospital and in-hospital settings. This noninvasive device may facilitate such advanced monitoring, yet there is still a need to show it in future studies in various clinical settings, evaluating its advantages and barriers.

## Funding support and author disclosures

Biobeat Technologies Ltd has provided the monitoring devices at no cost. Drs Goldstein, Ben Ishay, Fons, and Merin are employees of Biobeat Technologies Ltd. All other authors have reported that they have no relationships relevant to the contents of this paper to disclose.
