# Supplementary material for: Assessing Cardiac Flow Measurements Using a Noninvasive Photoplethysmography-Based Device Compared to Invasive Pulmonary Artery Catheter
Source: JACC Adv. 2025 Aug 22;4(9):102093. doi: 10.1016/j.jacadv.2025.102093 (PMC12398815; doi:10.1016/j.jacadv.2025.102093)
Supplement: Supplemental_Material [file mmc1.pdf]

SUPPLEMENTAL APPENDIX - Assessing Cardiac Flow Measurements Using a Noninvasive Photoplethysmography-Based Device Compared to Invasive Pulmonary Artery Catheter

SUPPLEMENTAL FIGURES LEGENDS

Supplemental Figure 1. Comparison of cardiac output measurements in normal weight individuals. Normal weight individuals based on the WHO definitions (BMI<25). A) Bland-Altman analysis between the PPG-based device and the Fick method; B) Bland-Altman analysis between the thermodilution method and the Fick method; C) Bland-Altman analysis between the PPG-based device and the thermodilution method; D) percentage of difference of the PPG-based device and Fick; E) percentage of difference of the thermodilution and Fick; F) percentage of difference of the PPG-based device and the thermodilution method; G) the correlation between the PPG-based device and the Fick method; H) the correlation between the thermodilution method and the Fick method; and I) the correlation between the PPG-based device and the thermodilution method. BMI, body mass index. CO, cardiac output. LOA, limit of agreement. PPG, photoplethysmography. TD, thermodilution.

Supplemental Figure 2. Comparison of cardiac output measurements in overweight individuals. Overweight individuals based on the WHO definitions (25≤BMI<30). A) Bland-Altman analysis between the PPG-based device and the Fick method; B) Bland-Altman analysis between the thermodilution method and the Fick method; C) Bland-Altman analysis between the PPG-based device and the thermodilution method; D) percentage of difference of the PPG-based device and Fick; E) percentage of difference of the thermodilution and Fick; F) percentage of difference of the PPG-based device and the thermodilution method; G) the correlation between the PPG-based device and the Fick method; H) the correlation between the thermodilution method and the Fick method; and

I) the correlation between the PPG-based device and the thermodilution method. BMI, body mass index. CO, cardiac output. LOA, limit of agreement. PPG, photoplethysmography. TD, thermodilution.

Supplemental Figure 3. Comparison of cardiac output measurements in obese individuals. Obese individuals based on the WHO definitions (30≤BMI). A) Bland-Altman analysis between the PPG-based device and the Fick method; B) Bland-Altman analysis between the thermodilution method and the Fick method; C) Bland-Altman analysis between the PPG-based device and the thermodilution method; D) percentage of difference of the PPG-based device and Fick; E) percentage of difference of the thermodilution and Fick; F) percentage of difference of the PPG-based device and the thermodilution method; G) the correlation between the PPG-based device and the Fick method; H) the correlation between the thermodilution method and the Fick method; and I) the correlation between the PPG-based device and the thermodilution method. BMI, body mass index. CO, cardiac output. LOA, limit of agreement. PPG, photoplethysmography. TD, thermodilution.

Supplemental Figure 4. **Comparison of cardiac output measurements in patients with Fitzpatrick 1-3.** A) Bland-Altman analysis of measurements between the PPG-based device and the Fick method; B) Bland-Altman analysis of measurements between the thermodilution and the Fick methods; C) Bland-Altman analysis between the PPG-based device and the thermodilution method; D) percentage of difference of the PPG-based device and Fick; E) percentage of difference of the thermodilution and Fick; F) percentage of difference of the PPG-based device and the thermodilution method; G) the correlation between the PPG-based device and the Fick method; H) the correlation between the thermodilution method and the Fick method; and I) the correlation between the PPG-based device and the thermodilution method. Type 1 - always burns, never tans, palest, can have freckles; Type 2 - usually burns, tans minimally, light-colored but darker than fair;

Type 3 - sometimes mild burn, tans uniformly, golden honey or olive. CO, cardiac output. LOA, limit of agreement. PPG, photoplethysmography. TD, thermodilution.

Supplemental Figure 5. **Comparison of cardiac output measurements in patients with Fitzpatrick 4-6.** A) Bland-Altman analysis of measurements between the PPG-based device and the Fick method; B) Bland-Altman analysis of measurements between the thermodilution and the Fick methods; C) Bland-Altman analysis between the PPG-based device and the thermodilution method; D) percentage of difference of the PPG-based device and Fick; E) percentage of difference of the thermodilution and Fick; F) percentage of difference of the PPG-based device and the thermodilution method; G) the correlation between the PPG-based device and the Fick method; H) the correlation between the thermodilution method and the Fick method; and I) the correlation between the PPG-based device and the thermodilution method. Type 4 - burns minimally, always tans well, moderate brown; Type 5 - very rarely burns, tans very easily, dark brown; and Type 6 - never burns, deeply pigmented dark brown to darkest brown. CO, cardiac output. LOA, limit of agreement. PPG, photoplethysmography. TD, thermodilution.

Supplemental Figure 6. Comparison of systemic vascular resistance measurements in normal weight individuals. Normal weight individuals based on the WHO definitions (BMI<25). A) Bland-Altman analysis between the PPG-based device and the Fick method; B) Bland-Altman analysis between the thermodilution method and the Fick method; C) Bland-Altman analysis between the PPG-based device and the thermodilution method; D) percentage of difference of the PPG-based device and Fick; E) percentage of difference of the thermodilution and Fick; F) percentage of difference of the PPG-based device and the thermodilution method; G) the correlation between the PPG-based device and the Fick method; H) the correlation between the thermodilution method and the Fick method; and I) the correlation between the PPG-based device and the thermodilution method. BMI, body mass index. LOA, limit of agreement. PPG, photoplethysmography. SVR, systemic vascular resistance. TD, thermodilution.

Supplemental Figure 7. Comparison of systemic vascular resistance measurements in overweight individuals. Overweight individuals based on the WHO definitions (25≤BMI<30). A) Bland-Altman analysis between the PPG-based device and the Fick method; B) Bland-Altman analysis between the thermodilution method and the Fick method; C) Bland-Altman analysis between the PPG-based device and the thermodilution method; D) percentage of difference of the PPG-based device and Fick; E) percentage of difference of the thermodilution and Fick; F) percentage of difference of the PPG-based device and the thermodilution method; G) the correlation between the PPG-based device and the Fick method; H) the correlation between the thermodilution method and the Fick method; and I) the correlation between the PPG-based device and the thermodilution method. BMI, body mass index. LOA, limit of agreement. PPG, photoplethysmography. SVR, systemic vascular resistance. TD, thermodilution.

Supplemental Figure 8. Comparison of systemic vascular resistance measurements in obese individuals. Obese individuals based on the WHO definitions (30≤BMI). A) Bland-Altman analysis between the PPG-based device and the Fick method; B) Bland-Altman analysis between the thermodilution method and the Fick method; C) Bland-Altman analysis between the PPG-based device and the thermodilution method; D) percentage of difference of the PPG-based device and Fick; E) percentage of difference of the thermodilution and Fick; F) percentage of difference of the PPG-based device and the thermodilution method; G) the correlation between the PPG-based device and the Fick method; H) the correlation between the thermodilution method and the Fick method; and I) the correlation between the PPG-based device and the thermodilution method. BMI, body mass index. LOA, limit of agreement. PPG, photoplethysmography. SVR, systemic vascular resistance. TD, thermodilution.

Supplemental Figure 9. **Comparison of systemic vascular resistance measurements in patients with Fitzpatrick 1-3.** A) Bland-Altman analysis of measurements between the PPG-based device and the Fick method; B) Bland-Altman analysis of measurements between the thermodilution and

the Fick methods; C) Bland-Altman analysis between the PPG-based device and the thermodilution method; D) percentage of difference of the PPG-based device and Fick; E) percentage of difference of the thermodilution and Fick; F) percentage of difference of the PPG-based device and the thermodilution method; G) the correlation between the PPG-based device and the Fick method; H) the correlation between the thermodilution method and the Fick method; and I) the correlation between the PPG-based device and the thermodilution method. Type 1 - always burns, never tans, palest, can have freckles; Type 2 - usually burns, tans minimally, light-colored but darker than fair; Type 3 - sometimes mild burn, tans uniformly, golden honey or olive. LOA, limit of agreement. PPG, photoplethysmography. SVR, systemic vascular resistance. TD, thermodilution.

Supplemental Figure 10. **Comparison of systemic vascular resistance measurements in patients with Fitzpatrick 4-6.** A) Bland-Altman analysis of measurements between the PPG-based device and the Fick method; B) Bland-Altman analysis of measurements between the thermodilution and the Fick methods; C) Bland-Altman analysis between the PPG-based device and the thermodilution method; D) percentage of difference of the PPG-based device and Fick; E) percentage of difference of the thermodilution and Fick; F) percentage of difference of the PPG-based device and the thermodilution method; G) the correlation between the PPG-based device and the Fick method; H) the correlation between the thermodilution method and the Fick method; and I) the correlation between the PPG-based device and the thermodilution method. Type 4 - burns minimally, always tans well, moderate brown; Type 5 - very rarely burns, tans very easily, dark brown; and Type 6 - never burns, deeply pigmented dark brown to darkest brown. LOA, limit of agreement. PPG, photoplethysmography. SVR, systemic vascular resistance. TD, thermodilution.

SUPPLEMENTAL FIGURE 1

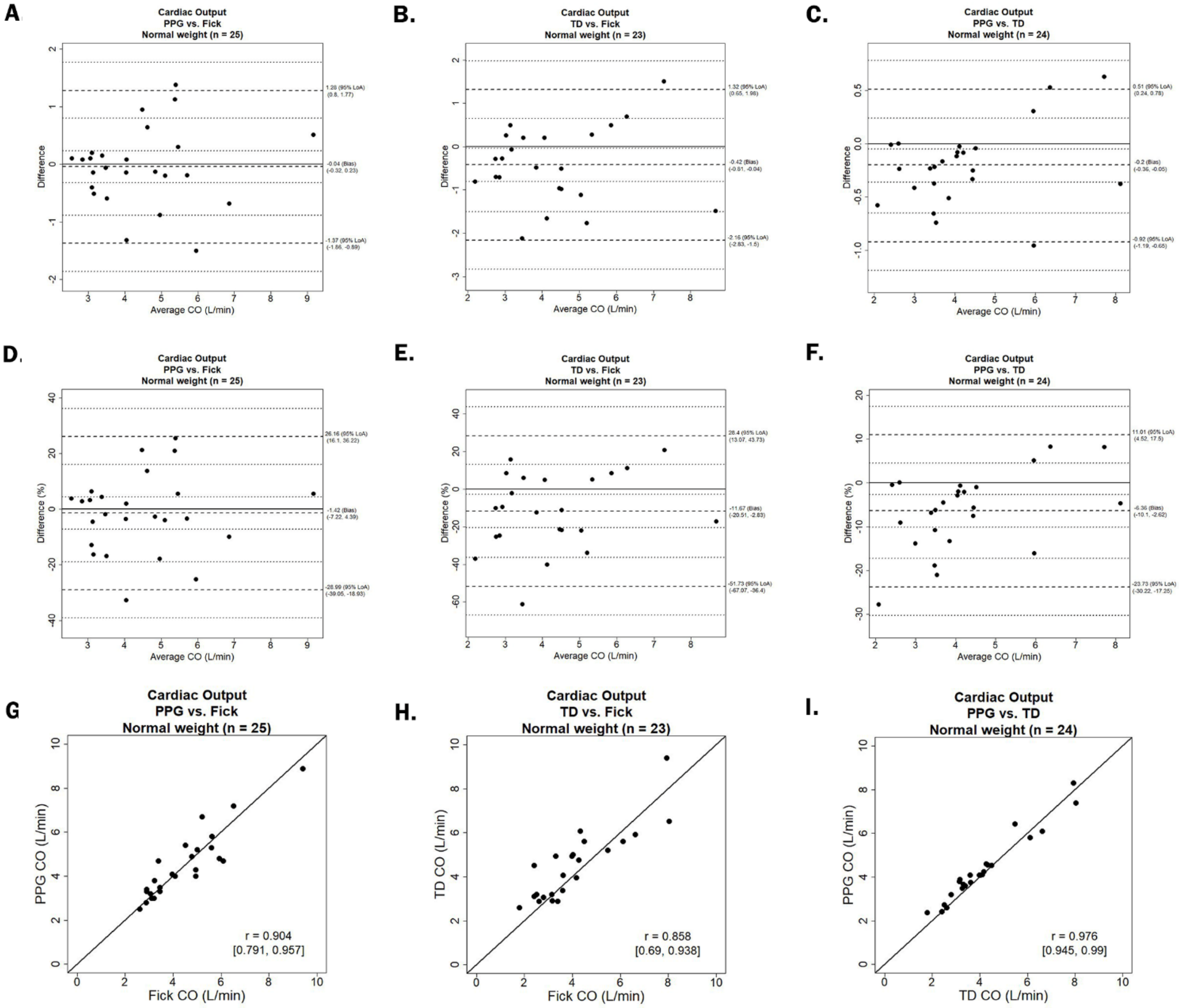

SUPPLEMENTAL FIGURE 2

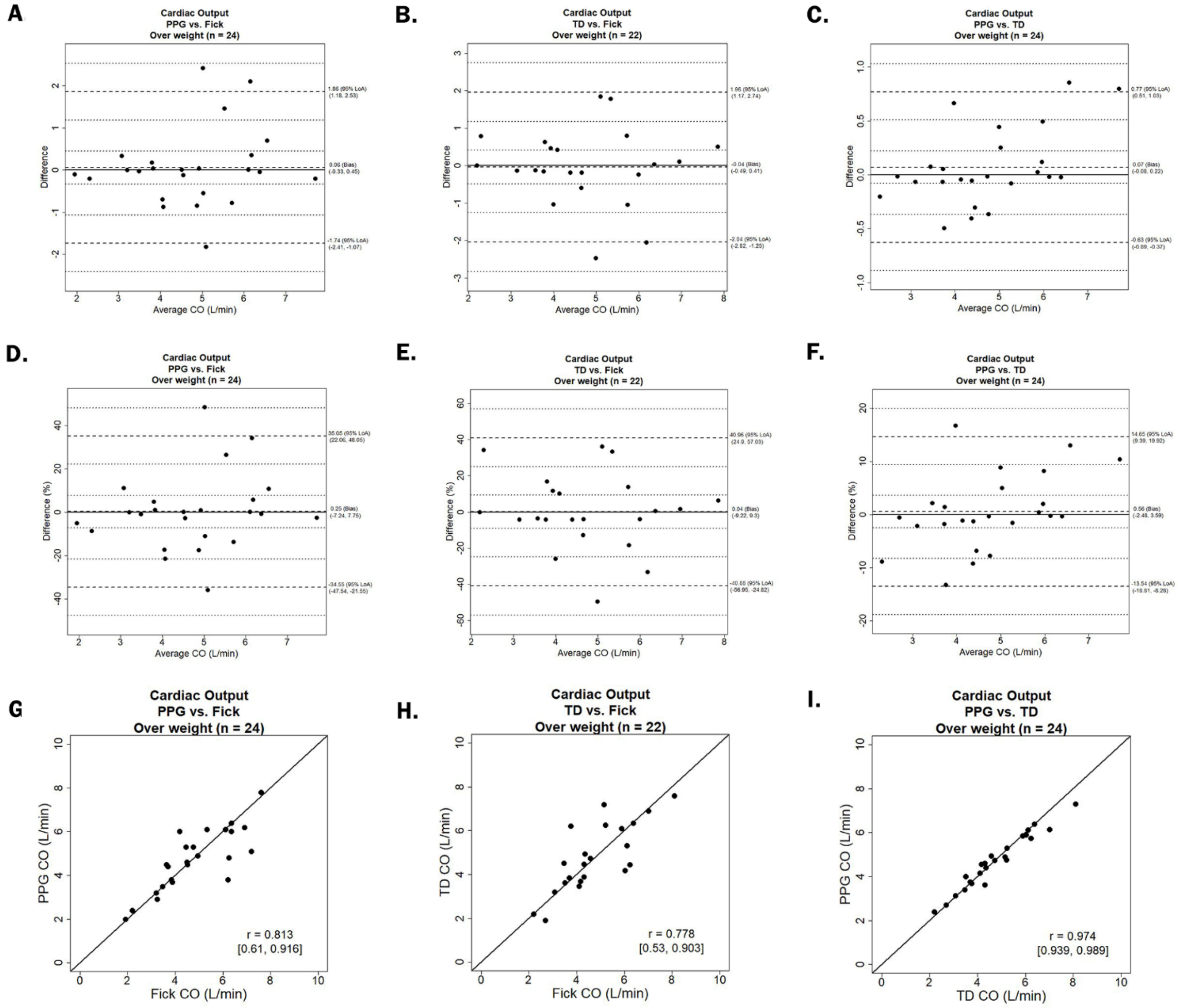

SUPPLEMENTAL FIGURE 3

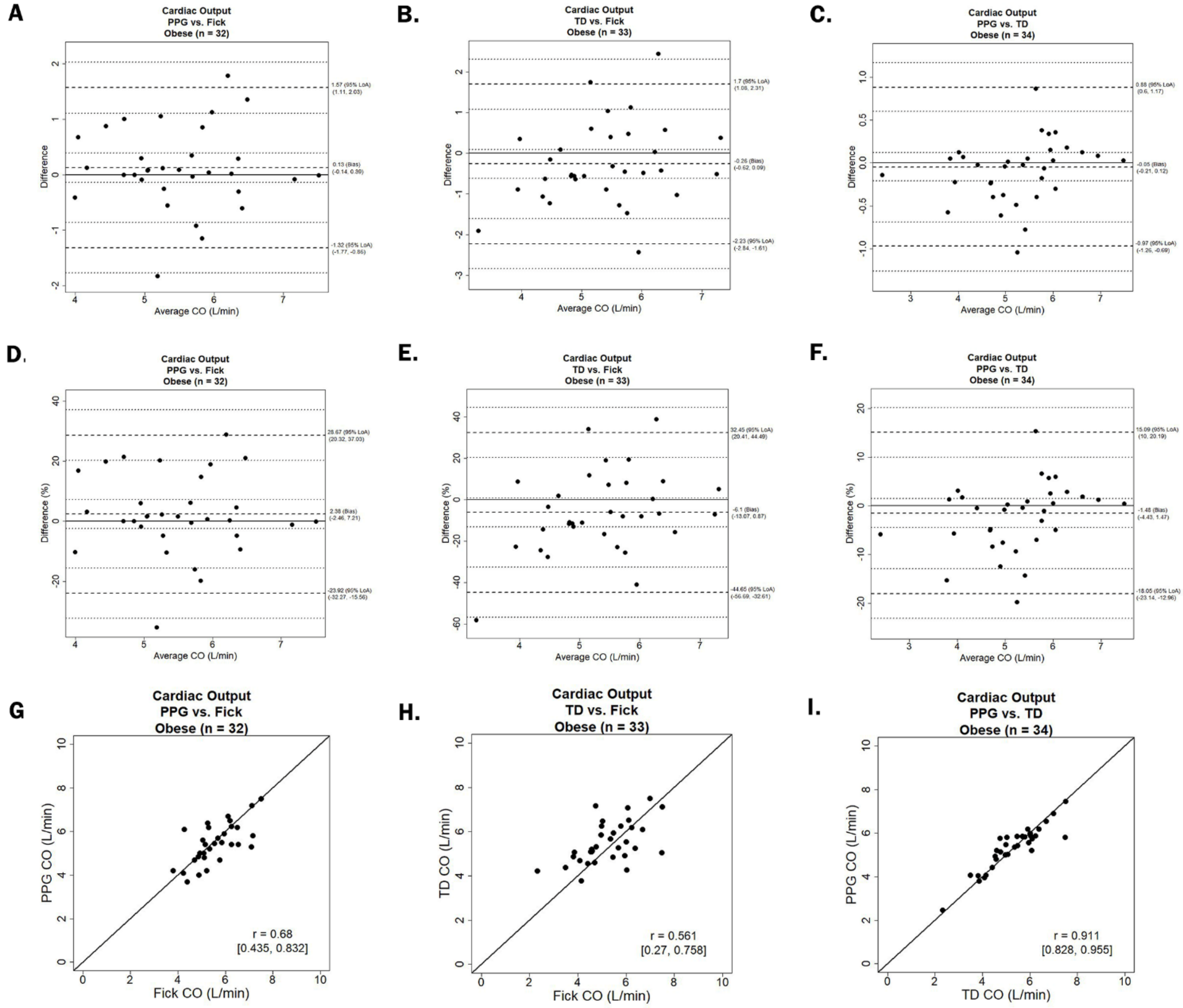

SUPPLEMENTAL FIGURE 4

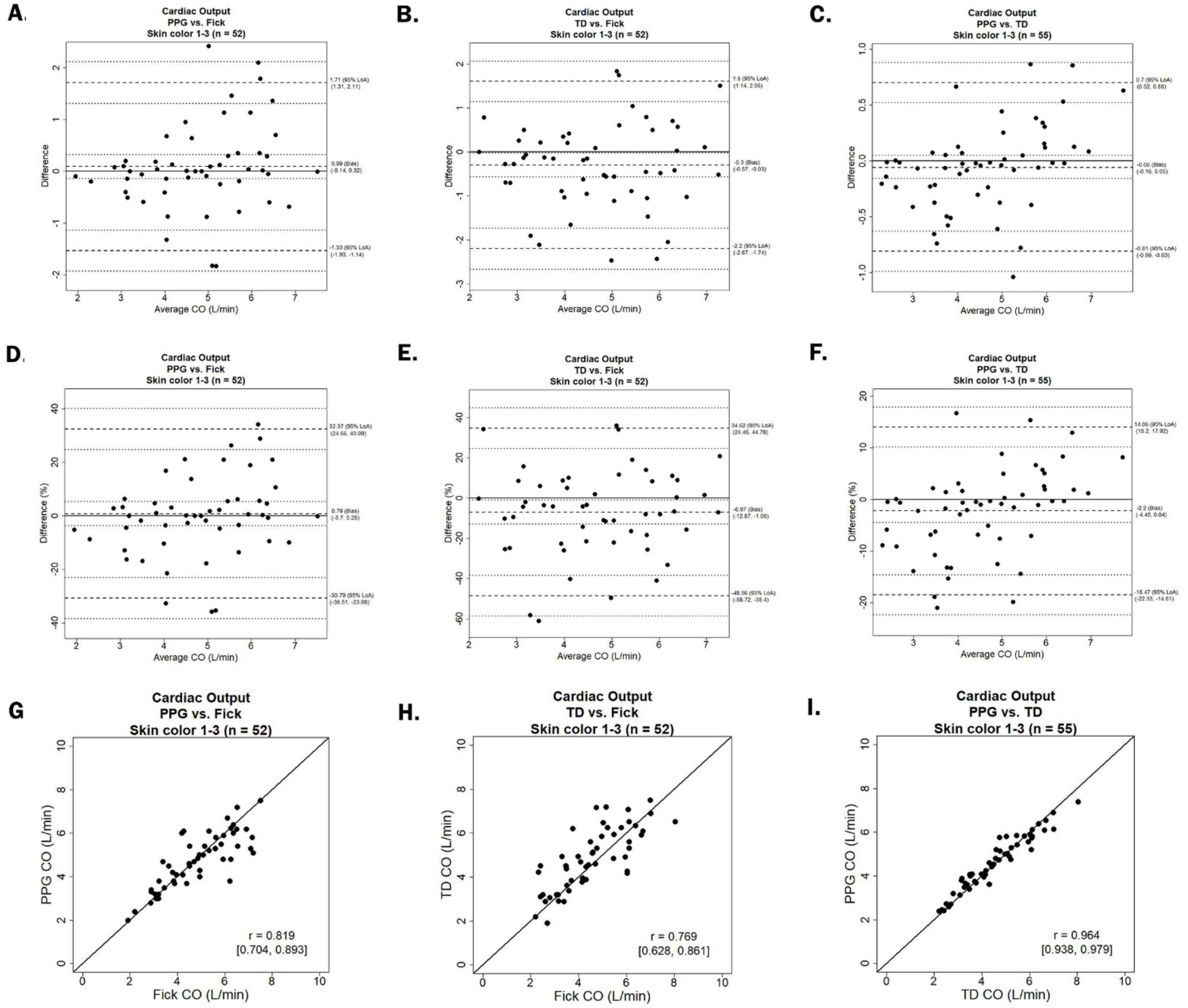

SUPPLEMENTAL FIGURE 5

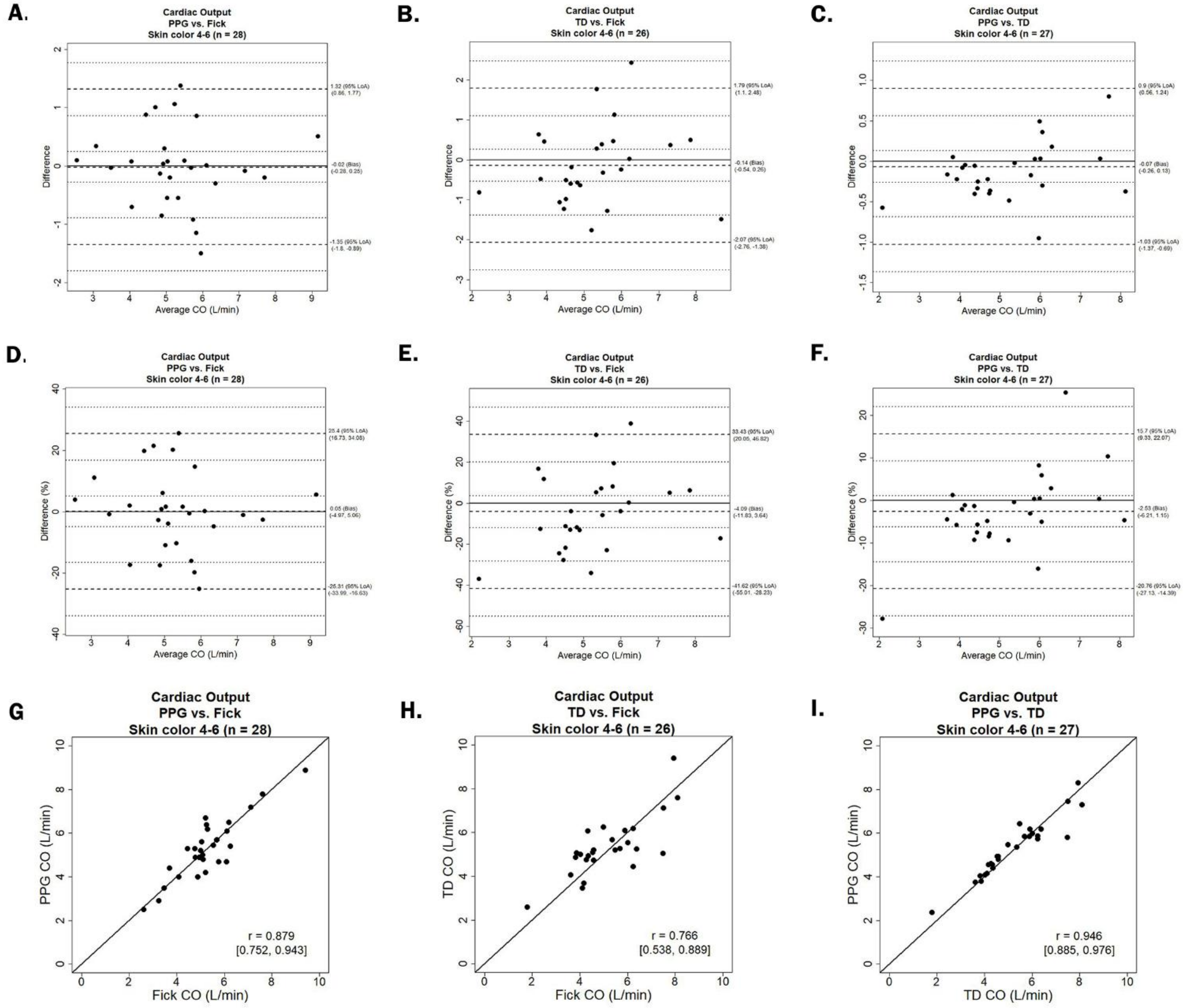

SUPPLEMENTAL FIGURE 6

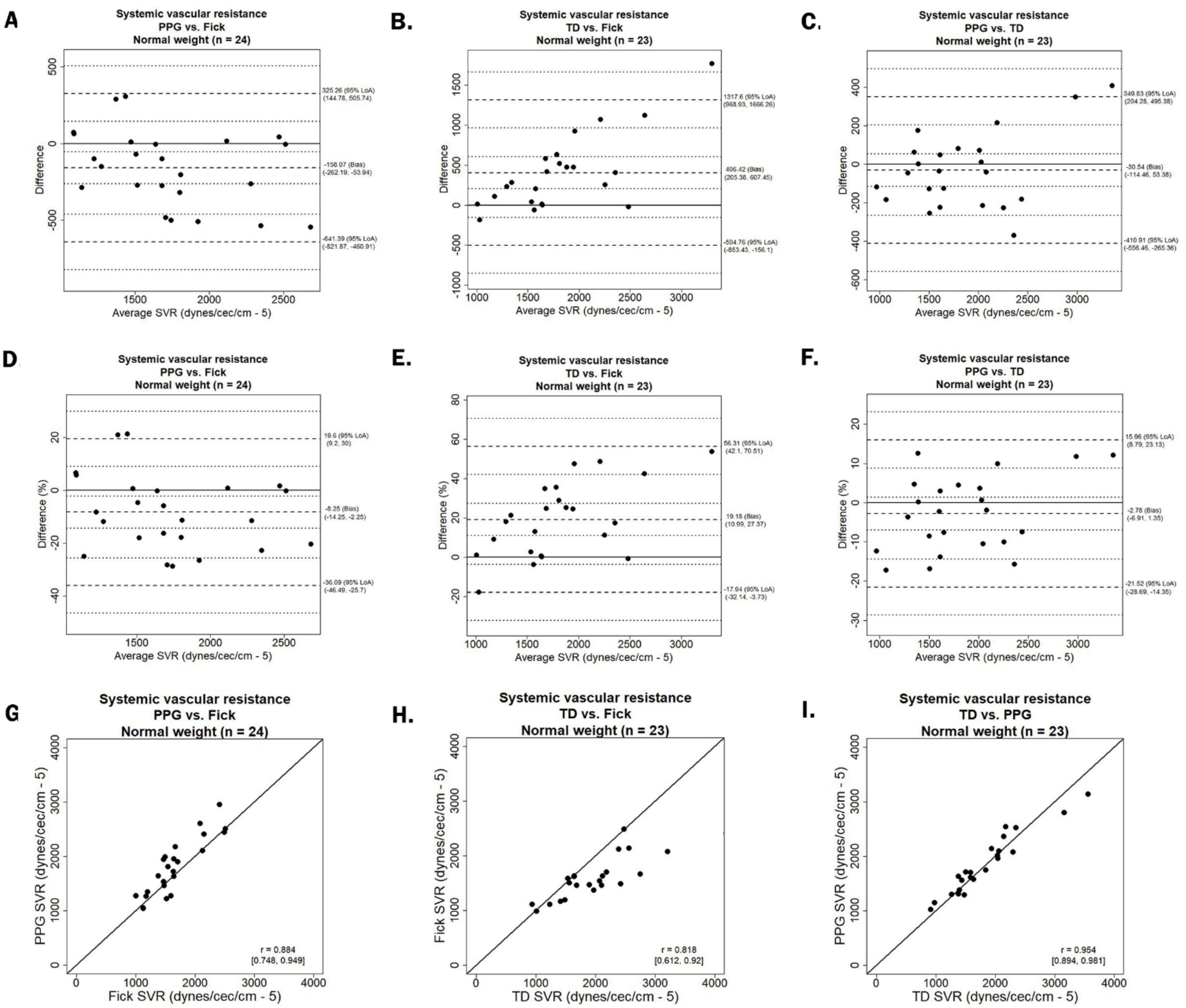

SUPPLEMENTAL FIGURE 7

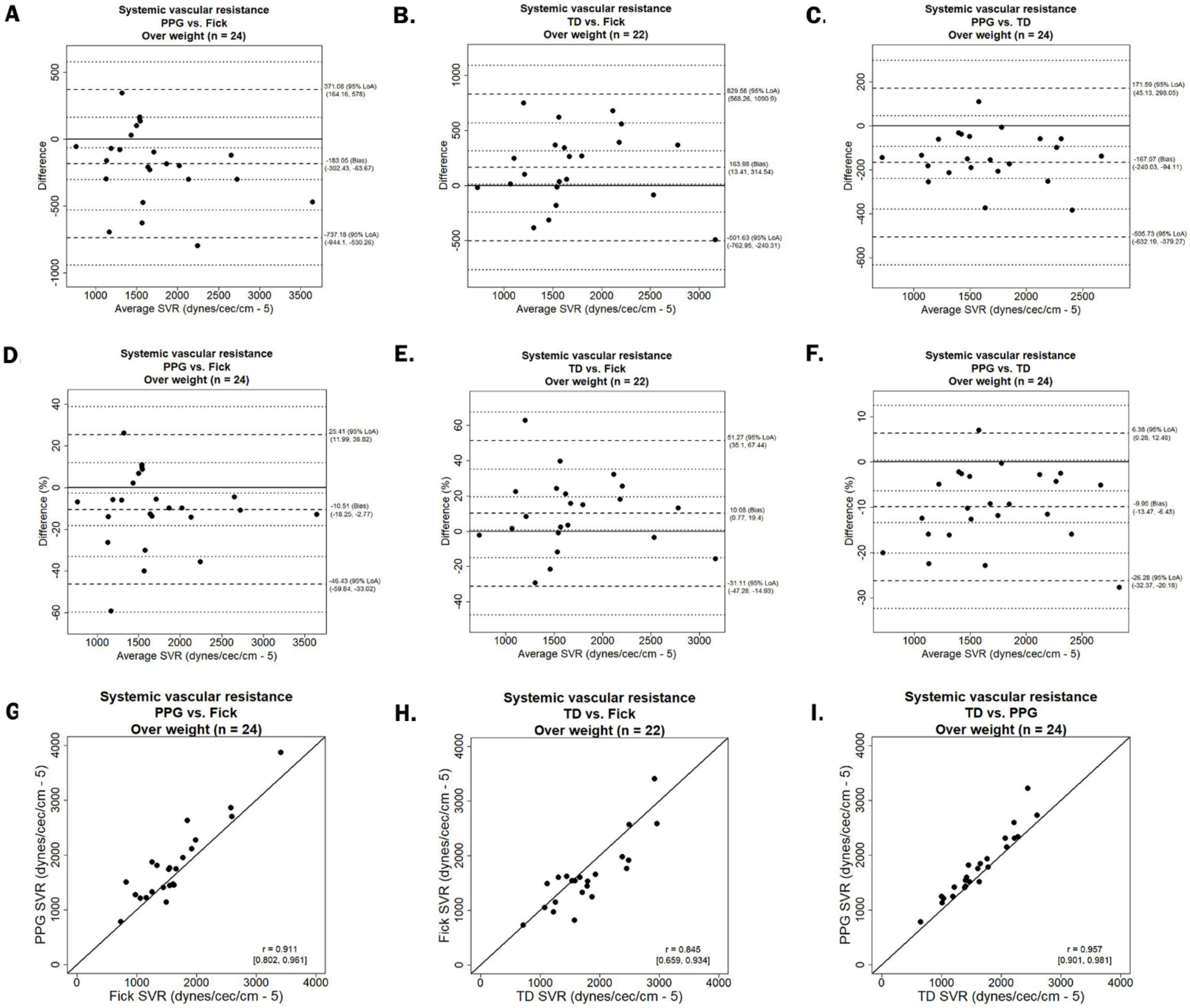

SUPPLEMENTAL FIGURE 8

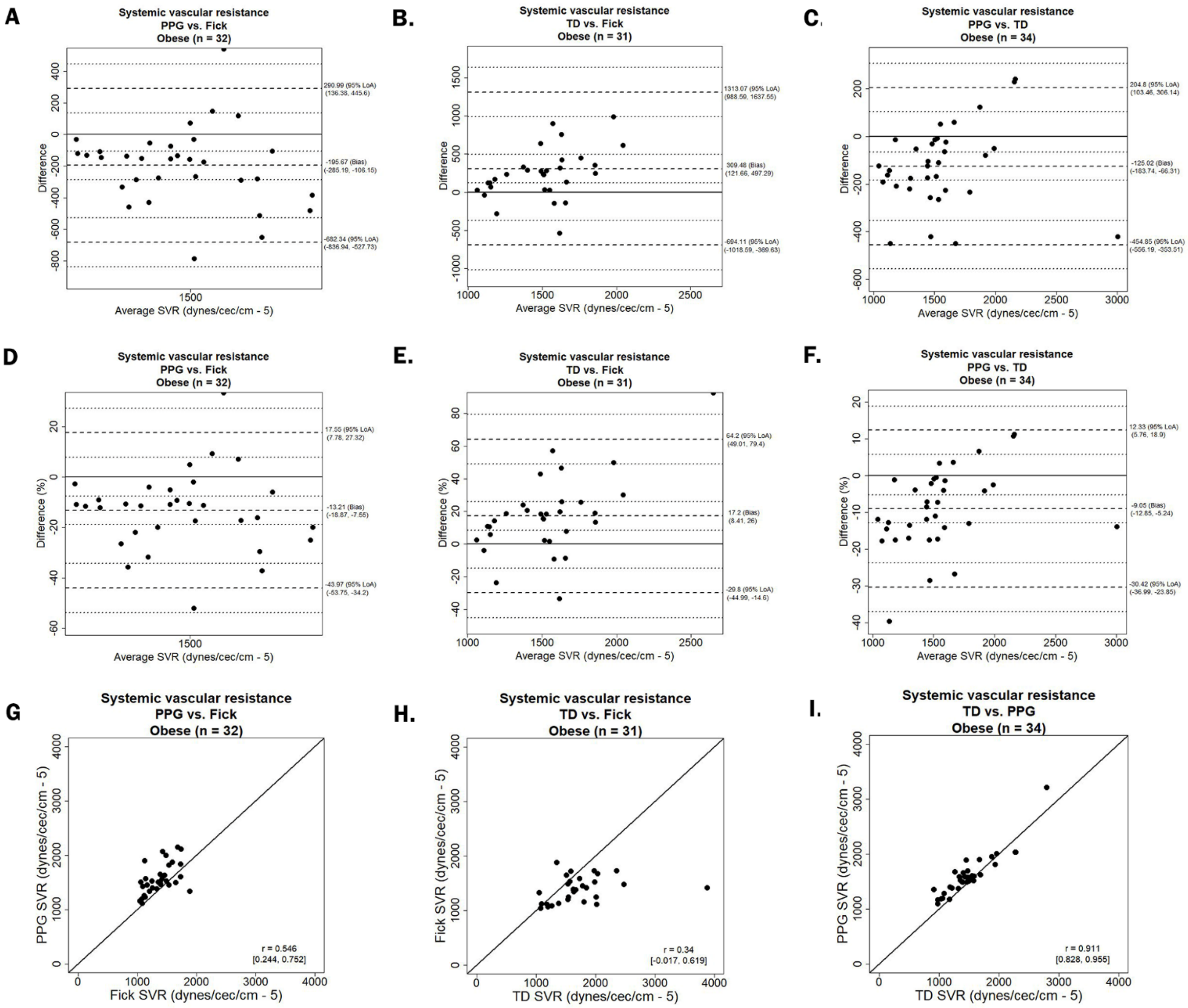

SUPPLEMENTAL FIGURE 9

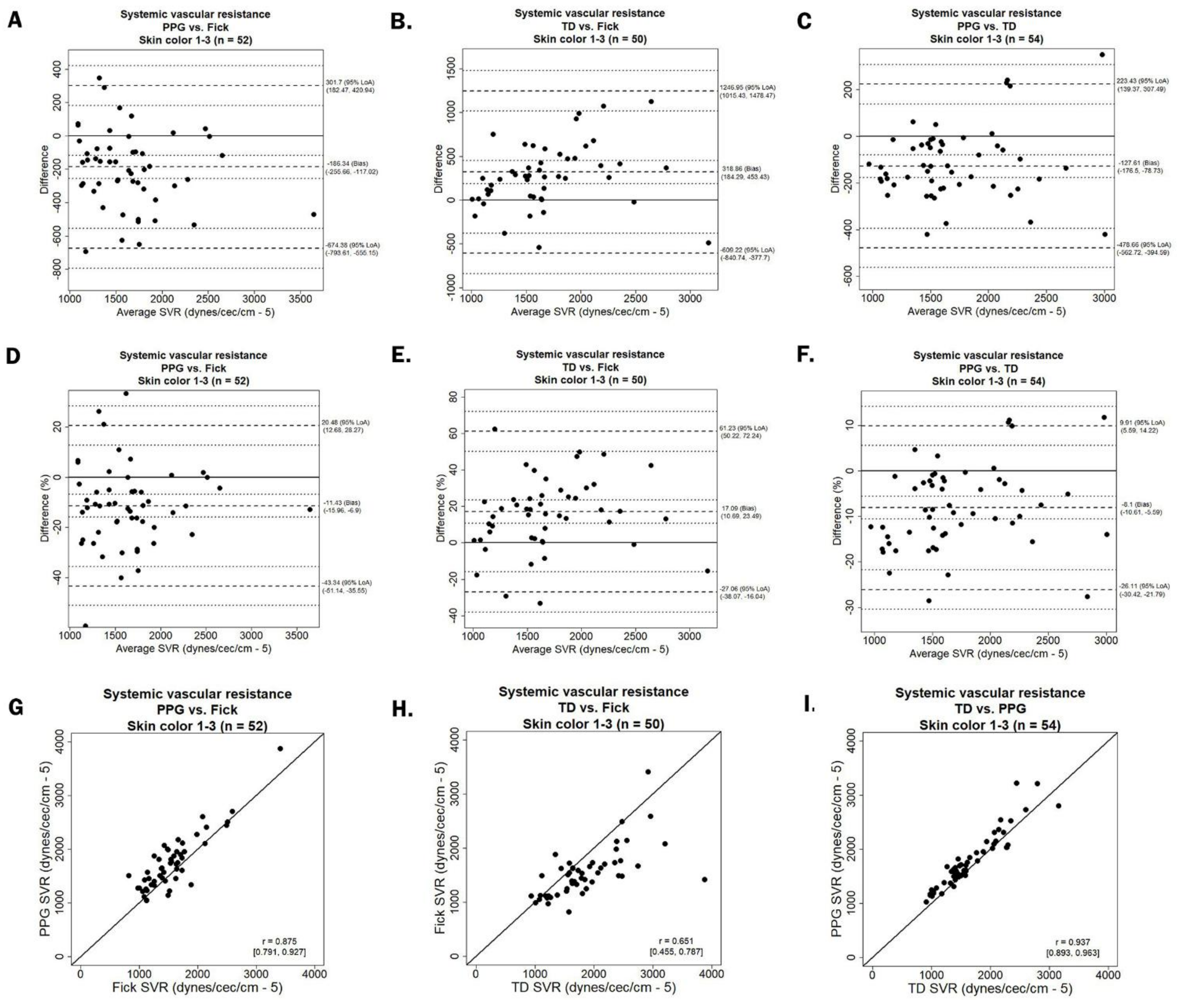

SUPPLEMENTAL FIGURE 10

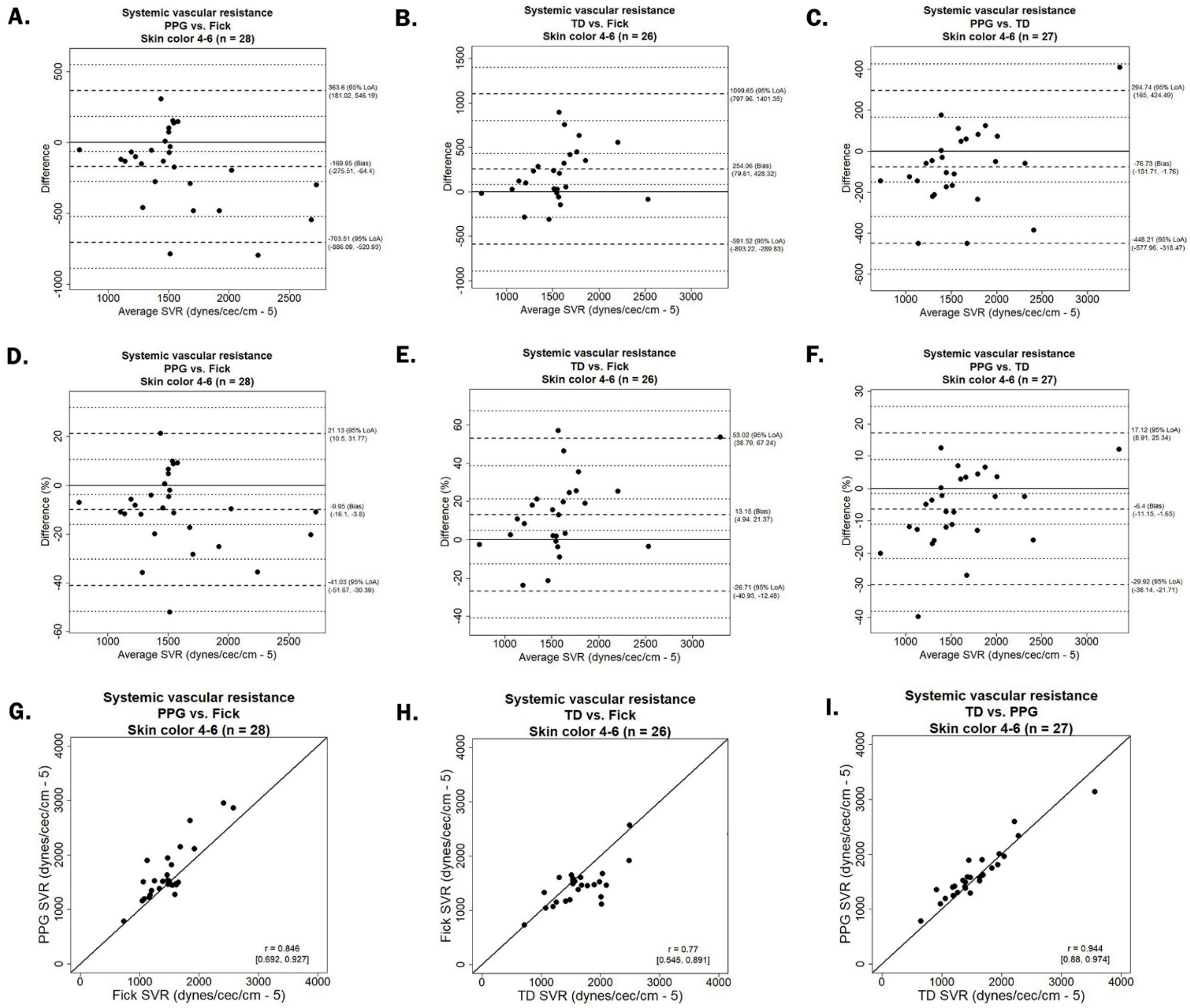

SUPPLEMENTAL TABLES:

Supplemental Table 1. Comparison of cardiac output measurement with the different modalities: descriptive values and agreement statistics. CI = confidence interval; IF = indirect Fick; LOA = limits of agreement; PPG = photoplethysmography; SD = standard deviation; TD = thermodilution.

**Measure**	**IF**	**PPG**	**TD**	
Mean ± SD (L/min)	5.0 ± 1.4	4.9 ± 1.3	4.9 ± 1.6	
**Absolute values (L/min)**		**Bias (95 % CI)**	**LOA (lower, upper)**	**Pearson *r* (95 % CI)**
PPG vs IF		−0.05 (−0.10, 0.20)	−1.4, 1.5	0.844 (0.767, 0.897)
TD vs IF		−0.20 (−0.40, 0.00)	−2.1, 1.6	0.775 (0.668, 0.851)
PPG vs TD		−0.06 (−0.15, 0.00)	−0.8, 0.7	0.959 (0.938, 0.974)
**Percentage difference (%)**				
PPG vs IF		−0.5 (−2.7, 3.8)	−28.6, 29.8	—
TD vs IF		−6.0 (−10.6, 1.3)	−46.1, 34.1	—
PPG vs TD		−2.3 (−4.2, −0.4)	−19.1, 14.5	—

Supplemental Table 2. Comparison of systemic vascular resistance measurement with the different modalities: descriptive values and agreement statistics. CI = confidence interval; IF = indirect Fick; LOA = limits of agreement; PPG = photoplethysmography; SD = standard deviation; TD = thermodilution.

**Measure**	**IF**	**PPG**	**TD**
Mean ± SD (dyn/s/cm^{-5})	1 720 ± 513	1 737 ± 511	1 793 ± 603

**Absolute values (dyn/s/cm^{-5})**	**Bias (95 % CI)**	**LOA (lower, upper)**	**Pearson *r* (95 % CI)**
PPG vs IF	–180.6 (–237.5, –123.6)	–681.9, 320.6	0.865 (0.796, 0.911)
TD vs IF	296.7 (192.1, 401.2)	–600.3, 1 193.7	0.687 (0.546, 0.790)
PPG vs TD	–110.6 (–151.1, –70.1)	–469.4, 248.1	0.936 (0.902, 0.959)
**Percentage difference (%)**			
PPG vs IF	–10.9 (–14.4, –7.3)	–42.3, 20.5	—
TD vs IF	15.7 (10.7, 20.7)	–26.8, 58.3	—
PPG vs TD	–7.5 (–9.7, –5.2)	–27.4, 12.3	—
